# Linear Regression Analysis of Sleep Quality in People with Insomnia in Wuhan City during the COVID-19 Pandemic

**DOI:** 10.1155/2023/6746045

**Published:** 2023-04-07

**Authors:** Hai-Tao Jin, Fei Wang, Wen Zhang, Qi-Lin Liu, Jing-Lan Zhang, Miao Yu, Zhen-Zhen Guo, Wei Pan

**Affiliations:** ^1^Department of Encephalopathy, The Traditional Chinese Medicine Hospital of Wuhan, Wuhan, Hubei 430014, China; ^2^Clinical College of Traditional Chinese Medicine, Hubei University of Chinese Medicine, Wuchang, Wuhan, Hubei 430065, China

## Abstract

**Objective:**

COVID-19 has evolved into a major global public health event. The number of people reporting insomnia is growing exponentially during the pandemic. This study aimed to explore the relationship between aggravated insomnia and COVID-19-induced psychological impact on the public, lifestyle changes, and anxiety about the future.

**Methods:**

In this cross-sectional study, we used the questionnaires from 400 subjects who were obtained from the Department of Encephalopathy of the Wuhan Hospital of Traditional Chinese Medicine between July 2020 and July 2021. The data collected for the study included demographic characteristics of the participants and psychological scales consisting of the Spiegel Sleep Questionnaire, the Fear of COVID-19 Scale (FCV-19S), the Zung Self-Rating Anxiety Scale (SAS), and the Zung Self-Rating Depression Scale (SDS). The independent sample *t*-test and one-way ANOVA were used to compare the results. Correlation analysis of variables affecting insomnia was performed using Pearson correlation analysis. The degree of influence of the variables on insomnia was determined using linear regression, and a regression equation was derived.

**Results:**

A total of 400 insomnia patients participated in the survey. The median age was 45.75 ± 15.04 years. The average score of the Spiegel Sleep Questionnaire was 17.29 ± 6.36, that of SAS was 52.47 ± 10.39, that of SDS was 65.89 ± 8.72, and that of FCV-19S was 16.09 ± 6.81. The scores of FCV-19S, SAS, and SDS were closely related to insomnia, and the influencing degree was in the following order: fear, depression, and anxiety (OR = 1.30, 0.709, and 0.63, respectively).

**Conclusion:**

Fear of COVID-19 can be one of the primary contributors to worsening insomnia.

## 1. Introduction

The novel coronavirus pneumonia (COVID-19) pandemic poses a grave threat to the physical and psychological health of people, causing panic among the global public [1]. The COVID-19 epidemic has been effectively contained due to the efforts of the Chinese government and health agencies. The epidemic prevention and control work in China has changed from emergency to the normalization phase [2–5]. Previous evidence has revealed that the COVID-19 epidemic will have an ongoing effect on humanity [6]. In the later stages of the epidemic, people have experienced psychological problems such as emotional instability, inattentiveness, and depression and reduced motivation.

Insomnia is a common clinical condition. Its etiology and pathogenesis are complex and may involve two aspects: on the one hand, it is associated with gender, age, genetic factors, income status, marital happiness, education level, and other vulnerability factors. On the other hand, it is associated with the sleep environment, sleep hygiene, psychological factors, and physical diseases [7]. It has been confirmed that medical personnel have higher detection rates of sleep problems, depression, and anxiety symptoms under the normalized COVID-19 epidemic prevention and control, at 40.4%, 25.9%, and 9.2%, respectively [7]. The impact of the pandemic on individual mental health is likely to persist for years after the pandemic [8]. However, the relationships among insomnia, depression, and anxiety during the pandemic remain unclear.

In this study, insomnia patients who were the residents of Wuhan city, the first place to report the outbreak of the COVID-19 epidemic, were recruited. We aimed to investigate the influencing factors such as fear of COVID-19, depression, and anxiety for insomnia among residents under normalized COVID-19 prevention and control. The data collected from this population may reflect the relationship between insomnia and the psychological state more comprehensively and therefore may provide better reference for the development of mental health management strategies and targeted psychological interventions.

## 2. Materials and Methods

### 2.1. Participants

In this study, we used questionnaires to investigate patients at the Department of Encephalopathy of the Wuhan Hospital of Traditional Chinese Medicine from July 2020 to July 2021. The inclusion criteria were as follows: (1) aged between 18 and 80 years old, (2) diagnosed with chronic insomnia with a duration of ≥6 months, and (3) able to understand the questionnaire and willing to cooperate. The diagnosis of insomnia was made in accordance with the Chinese Classification of Mental Disorders (CCMD-3-R) [9]. Participants who had severe mental illnesses or cardiovascular, cerebrovascular, or other serious diseases were excluded. Researchers collected and uniformly recorded the data. A total of 439 questionnaires were received. After excluding 39 invalid questionnaires, a total of 400 questionnaires were included in the final analysis, with an effective recovery rate of 91.12%. Informed consent forms were obtained from all participants. The study was approved by the Ethics Committee of the Wuhan Hospital of Traditional Chinese Medicine.

### 2.2. Methods

#### 2.2.1. Research Tools

Questionnaires used in the study were as follows: (1) For general demographic data on respondents, the questionnaire included details about their sociodemographic characteristics, including age, gender, marital status, education level, and occupation. (2) As anxiety, depression, and fear are related to insomnia and may be confounding factors, participants were screened for anxiety and depression with the Zung Self-Rating Anxiety Scale (SAS) and the Zung Self-Rating Depression Scale (SDS), respectively. The Spiegel Sleep Questionnaire was used to assess the severity of insomnia, and the Fear of COVID-19 Scale (FCV-19S) was used to evaluate the fear of COVID-19.

The Spiegel Sleep Questionnaire is an internationally accepted sleep scale, which assesses 6 domains: sleep latency, sleep duration, night waking occurrences, sleep depth, night-time dreams, and a feeling of being awake. Each item is scored according to the following scores: 0, 1, 3, 5, and 7, and the cumulative scores of each domain are the total scores of the questionnaire. The higher the scores, the more severe the insomnia. The evaluation is as follows: mild insomnia if the total score is ≥12 points, moderate insomnia if the total score is ≥18 points, and severe insomnia if the total score is ≥24 points. Cronbach's *α* coefficient of the Spiegel Sleep Questionnaire is 0.868. FCV-19S is a reliable and effective tool for assessing the level of fear of COVID-19 and consists of 7 items such as the level of understanding of the epidemic, the level of concern about the epidemic, and the level of panic about the epidemic. The response for each item on FCV-19S is rated from 1 (strongly disagree) to 5 (strongly agree) on a Likert scale. The total score is the sum of scores of all items, ranging from 7 to 35, with higher scores indicating greater fear of COVID-19 [10]. The scale had good reliability in this study, with Cronbach's *α* coefficient of 0.92.

Zung SAS is used to assess the severity of an individual's anxiety status [11]. The scale consists of 20 items, and each item is scored on four levels. In accordance with Chinese standards, a score of less than 50 indicates no anxiety symptoms, a score between 50 and 59 indicates mild anxiety, a score between 60 and 69 indicates moderate anxiety, and a score of over 69 indicates severe anxiety. In this study, Cronbach's *α* coefficient of SAS was 0.770.

Zung SDS is used to assess the severity of an individual's depression status [12]. The scale consists of 20 items and is scored on four levels. As per Chinese norms, a total score of <53 indicates no depression, a score between 53 and 62 indicates mild depression, a score between 63 and 72 indicates moderate depression, and a score ≥73 points indicates severe depression. In this study, Cronbach's *α* coefficient of SDS was 0.794.

## 3. Statistical Analysis

SPSS 25.0 software was used for statistical analysis. The demographic variables of the participants were descriptively analyzed, and the participants' ages and scale scores were calculated with a mean ± SD $\left(\bar{X}\;\pm \;S\right)$. The independent sample *t*-test and one-way ANOVA were adopted for comparisons between groups. Correlation analysis of variables affecting insomnia was performed using Pearson correlation analysis. We determined the degree of influence of certain variables on insomnia using linear regression and derived a regression equation.

## 4. Results

### 4.1. Demographic Characteristics

A total of 400 people participated in the study, with an average age of 45.75 ± 15.04 years. There were 185 males (46.3%) and 215 females (53.8%). Of the 400 participants, 305 were married (76.3%), 83 were unmarried (20.8%), and the remaining 12 were divorced or widowed (3.1%). There were 108 manual workers (27%), 155 clerks (38.8%), 48 students (12%), and 89 retirees or unemployed people. There were 165 participants with a high school degree or less (41.3%) and 235 with a university degree or above (58.7%) (Table 1).

### 4.2. Physiological Characteristics of Sleep of People with Insomnia

In terms of sleep time, the average bedtime was 22.34 ± 6.04. Among the study participants, 130 participants went to bed at 11 p.m. (32.5%), accounting for the largest proportion, while only 2 people went to bed at 1 a.m. (0.5%), making up the least percentage. The average wake-up time was 6.34 ± 0.94, and the number of times they woke during sleep was 2.19 ± 1.31 (Table 2).

The average score of the Spiegel Sleep Questionnaire in people with insomnia was 17.29 ± 6.36, that of SAS was 52.47 ± 10.39, that of SDS was 65.89 ± 8.72, and that of FCV-19S was 16.09 ± 6.81 (Table 3).

### 4.3. Test of Demographic Differences in People with Insomnia

The independent sample *t*-test and one-way ANOVA were used to examine the differences in demographic factors in people with insomnia, and the results are displayed in Table 4. Age, marital status, and occupation were significantly different demographic factors.

### 4.4. Correlation Analysis of the Variates

Pearson correlation analysis showed that the independent variables, namely, scores on FCV-19S (fear of COVID-19), SAS (anxiety), and SDS (depression), were significantly correlated with the dependent variable, namely, the Spiegel Sleep Questionnaire scores (insomnia) (*P* < 0.01). Also, the comparative analysis between the three variables showed a significant correlation (*P* < 0.01) (Table 5).

### 4.5. Linear Regression Analysis of Impact Factors

1. The linear regression model fitted well. The R^2^ value was 0.39, which is more than 0.3, indicating that the results of this study could truly and reliably reflect the influence of FCV-19S, SAS, SDS, age, occupation, and marital status on the degree of insomnia (Spiegel Sleep Questionnaire scores).
2. There was no multicollinearity between the three independent variables, with all variance inflation factor (VIF) values less than 5.
3. The regression equation was significant, *F* = 84.34, *P* < 0.001, indicating that at least one of the six independent variables could significantly affect sleep quality.
4. FCV-19S, anxiety, and depression had a significant positive effect on sleep quality (*β* = 1.30 > 0, *P* < 0.00; *β* = 0.63 > 0, *P* < 0.05; and *β* = 0.70 > 0, *P* < 0.05; respectively), while age had a significant negative effect on sleep quality (*β* = −0.005 < 0, *P* < 0.05). Moreover, marital status had a significant positive effect on sleep quality (*β* = 0.16 > 0, *P* < 0.016), while occupation had no correlation with insomnia (*P* > 0.05).

We derived the following regression equation between the variables: insomnia degree = 0.91 + 1.30 *∗* FCV-19S + 0.63 *∗* SAS + 0.70 *∗* SDS − 0.005 *∗* age + 0.16 marital status (Table 6).

## 5. Discussion

Insomnia is a common clinical disorder with multiple factors affecting its occurrence, including both environmental and behavioral factors. In this study, we found that the fear of COVID-19 is one of the primary contributors to worsening insomnia.

Major epidemic events may affect the factors influencing mental health [13]. For instance, the global coronavirus epidemic in 2003 caused mental health complications in 14%–61% of infected people, and 14.8%–76.9% people developed mental health problems after the infection [14]. Similarly, a study of 42 COVID-19 patients in the United Kingdom showed that more than one-third of patients had olfactory deficits and taste disturbances [15]. A higher proportion of insomnia cases were also observed in patients infected with COVID-19 [16]. These results suggest that insomnia is related to psychosomatic and socio-environmental factors related to COVID-19.

Some indicators of immune function respond to insomnia with impaired immune function [17]. The main cytokines involved in the regulation of sleep-wake rhythm are tumor necrosis factor (TNF), interferon, and interleukin (IL) [18]. Clinical observations of the normal sleep-wake cycle showed that proinflammatory cytokines increase in early sleep and that there is an increase in immune cells and anti-inflammatory cytokines during the daytime [19]. Moreover, immune function is significantly decreased in patients with generalized anxiety with sleep disorder compared to those with generalized anxiety and no sleep disorder [20]. The reason for this may be that insomnia, as a chronic stress, has a significant impact on the brain function and nervous system of the body, leading to dysfunction of 5-HT, which affects the homeostasis of the body. These findings revealed the involvement of neuro-endocrine-immune mechanisms in insomnia.

Cytokine storms in COVID-19 are closely associated with organ dysfunction, which can further lead to olfactory deficits or insomnia [21]. Cazzolla et al. suggested that proinflammatory cytokines, specifically TNF-*α*, may contribute to COVID-19-induced olfactory deficits and insomnia [22]. Another proinflammatory cytokine, IL-6, increases in cases with olfactory deficits [23, 24]. The process through which these cytokines produce olfactory deficit or insomnia is incomprehensible. Henkin et al. suggested that this effect may be caused by the peripheral or central action of cytokines due to viral infection [25]. The above data imply a correlation between insomnia and inflammatory cytokines related to COVID-19.

The risk of anxiety, depression, and insomnia increases with psychosomatic changes [6]. Available evidence suggests that some of the measures taken to curb the epidemic may harm vulnerable populations, especially those with mental or physical health problems and mental illness [26, 27]. COVID-19 isolation may increase anxiety and depression in people coping with mental or physical problems due to the lack of access to mental health support and positive activities. These results imply the effect of the COVID-19 epidemic on insomnia from a psychosomatic perspective.

The outbreaks of highly contagious diseases can bring associated psychiatric symptoms that suggest COVID-19 is a stressful life event and a leading cause of physical and mental disorders. For instance, the prevalence of anxiety, depression, and insomnia was much higher during the outbreak and remission phases of the COVID-19 outbreak in Hubei Province, China, in 2020 [7]. Moreover, during the COVID-19 pandemic, the relationship between mental health-related factors in 1,100 German study participants with insomnia showed that the prevalence of insomnia, anxiety, and depression was 19.5%, 4.8%, and 6.6%, respectively [28]. The mean and median insomnia severity index (ISI) scores, patient health questionnaire-4 (PHQ-4) scores, PHQ-2 scores, and generalized anxiety disorder (GAD-2) scores were lower in the noninsomnia group than those in the insomnia group, while the mean and median scores of “life quality” and “health quality” were higher and statistically significant (*P* < 0.05). Pearson's correlation analysis showed that ISI scores were positively correlated with PHQ-2 scores (*P* < 0.001), GAD-2 scores (*P* < 0.001), and PHQ-4 scores (*P* < 0.001). This study confirmed that Germans were affected by insomnia, anxiety, and depression during the COVID-19 pandemic more than ever before. Anxiety and depression symptoms had an effect on each other and were more severe in the insomnia group than those in the noninsomnia group. In a study on COVID-19 and factors such as anxiety, depression, and insomnia in Iran, among the 675 people with insomnia, the average age was 40.28 ± 11.15 years and the prevalence of difficulty in initiating sleep (DIS), difficulty in maintaining sleep (DMS), and early morning awakening (EMA) was 91.4%, 86.7%, and 77%, respectively [29]. DIS, DMS, and EMA were more common in patients with depression and anxiety. The Fear of COVID-19 Scale (FCV-19S) scores were higher in patients with more severe DIS, DMS, and EMA types (*P* < 0.001). FCV-19 was a risk factor for all insomnia patterns (DIS, DMS, and EMA; OR = 1.19, 1.12, and 1.02, respectively). Nevertheless, among demographic factors, it is believed that occupation is also a risk factor for insomnia, especially for those who are self-employed, which is different from this study. The reason could be that health precautions and lockdowns may have had a greater impact on their business.

In this study, we focused on the impacts of fear, anxiety, depression, and other factors on people with insomnia in Wuhan city, China, during the COVID-19 epidemic. Our data revealed that the average score of the Spiegel Sleep Questionnaire in people with insomnia was 17.29 ± 6.36, that of SAS was 52.47 ± 10.39, that of SDS was 65.89 ± 8.72, and that of FCV-19S was 16.09 ± 6.81. Most people reported mild to moderate insomnia. The relationship between self-reported anxiety, depression, and insomnia with respect to regular COVID-19 prevention and control measures among residents in Wuhan city changed after the addition of the variable of fear of COVID-19. The factors that were previously thought to affect insomnia, anxiety, and depression were different in this study. For example, the risk factors for insomnia were advanced age, female, divorce, unemployment, and higher education. The linear regression analysis showed that fear of COVID-19 was the biggest influencing factor of insomnia among these four factors followed by depression, anxiety, and marital status. Also, the younger the age is, the more likely the person was anxious, had insomnia, and was fearful. The above findings prove the relationship between fear and anxiety, depression, and insomnia. Fear affects the internal balance between mind and body, which leads to mind-body reactions such as stress, depression, anxiety, insomnia, or various physical problems. The wider community impact on the mental health of people and vulnerable populations will continue for a long time even after the COVID-19 pandemic ends. Therefore, we believe that the fear of COVID-19 is a risk factor for all types of insomnia. Lack of sleep may in turn lead to aggravation of negative emotions such as stress, anxiety, and worry, which creates a vicious cycle and leaves people with insomnia in a state of fear, anxiety, and depression for a long time.

As for the limitations of this study, first, it is a cross-sectional and self-reported survey. Respondents of different cultural levels and ages had different understandings of the information being collected, so responses may be biased, which could affect the accuracy of the information. Second, the participants of this study were limited to the people with insomnia in Wuhan city, and the sample size was relatively small. The survey did not target specific groups, and this study offers only a general overview.

In conclusion, our study, for the first time, reported the influencing factors for insomnia in residents of Wuhan city, China, where COVID-19 was first reported. Our results show that fear of COVID-19 is one of the primary contributors to increased insomnia. These findings suggest that psychological interventions should be part of the public health response following a major epidemic and must include various intervention strategies, address different intervention groups, and have diverse intervention forms. It is also important to let the public fully understand the rationale behind different policies during different phases of an epidemic, thus eliminating policy blind spots. Government agencies play a role in dispelling public panic by releasing information in a timely manner about the epidemic through official media; at the same time, at different phases of the epidemic, prevention and control policies must be adjusted to develop the economy and reduce economic pressure on the public.

## Figures and Tables

**Table 1:** Demographic characteristics of the participants.

	Category	Number	Percentage
Gender	Male	185	46.3
	Female	215	53.8

Age	18–29 years old	79	19.8
	30–49 years old	147	36.8
	50–69 years old	148	37
	70 years old and above	26	6.5

Marital status	Unmarried	83	20.8
	Married	305	76.3
	Divorced or widowed	12	3.1

Occupation	Manual worker	108	27
	Clerk	155	38.8
	Student	48	12
	Retiree or unemployed	89	22.3

Educational level	High school degree or less	165	41.3
	University degree or above	235	58.7

**Table 2:** Average bedtime, wake-up time, and number of awakenings.

	*N*	Value
Average bedtime	400	22.34 ± 6.04
Wake-up time	400	6.34 ± 0.94
Number of awakenings	400	2.19 ± 1.31

**Table 3:** Scores of different scales.

	Case	Average value	Standard deviation
Spiegel Sleep Questionnaire score	400	17.29	6.36
SAS score	400	52.47	10.39
SDS score	400	65.89	8.72
FCV-19S score	400	16.09	6.81

**Table 4:** Difference analysis of the demographic characteristics.

Variables	Group	Case	Spiegel Sleep Questionnaire score $\left(\bar{X}\pm S\right)$	*T*/*F*	*P*
Gender	Male	185	17.19 ± 6.38	−0.27	0.61
	Female	215	17.37 ± 6.36		

Age	18–29 years old	79	14.30 ± 6.77	9.767^*∗∗*^	0.00
	30–49 years old	147	17.71 ± 6.23		
	50–69 years old	148	18.75 ± 5.83		
	70 years old and above	26	15.65 ± 5.69		

Educational level	High school degree or less	165	17.55 ± 6.66	1.77	0.10
	University degree or above	235	18.76 ± 5.80		

Marital status	Unmarried	83	14.92 ± 6.97	7.51^*∗*^	0.001
	Married	305	17.92 ± 6.021		
	Divorced or widowed	12	17.58 ± 7.26		

Occupation	Manual worker	108	18.88 ± 5.96	5.86^*∗∗*^	0.00
	Clerk	155	17.46 ± 6.072		
	Student	48	13.44 ± 7.11		
	Retiree or unemployed	89	17.85 ± 6.32		

*Note.* ^*∗*^ *P* < 0.05; ^*∗∗*^*P* < 0.01.

**Table 5:** Correlation analysis between variables.

	FCV-19S	Spiegel	SAS	SDS
FCV-19S	1			
Spiegel	0.62^*∗∗*^	1		
SAS	0.36^*∗∗*^	0.47^*∗∗*^	1	
SDS	0.56^*∗∗*^	0.68^*∗∗*^	0.16^*∗∗*^	1

*Note.* ^*∗∗*^ *P* < 0.01 (two-tailed), significant correlation.

**Table 6:** Regression analysis of impact factors.

Model	Unstandardized coefficients		Standardized coefficients	*t*	Significance	VIF
	*B*	Standard error	Beta			
Constant	0.91	0.31	—	2.90	0.004	—
FCV-19S	1.30	0.08	0.61	15.14	0.000	1.06
SAS	0.63	0.08	0.031	0.76	0.041	1.04
SDS	0.70	0.10	0.039	0.96	0.032	1.03
Age	−0.005	0.06	−0.00	−0.08	0.022	1.71
Marital status	0.16	0.09	0.08	1.68	0.016	1.60
Occupation	−0.02	0.03	−0.03	−0.86	0.385	1.01
*R* ^2^				0.39		
*F*				43.15		
*P*				<0.00		

Dependent variable: Spiegel Sleep Questionnaire scores. Predictor variables: constant, occupation, marital status, age, FCV-19S scores, SAS scores, and SDS scores.

## Data Availability

All data generated or analyzed during this study are included in this article. Further enquiries can be directed to the corresponding author.
